# Genotype and clinical characteristics of patients with Wolfram syndrome and WFS1-related disorders

**DOI:** 10.3389/fgene.2023.1198171

**Published:** 2023-06-21

**Authors:** Evan M. Lee, Megha Verma, Nila Palaniappan, Emiko M. Pope, Sammie Lee, Lindsey Blacher, Pooja Neerumalla, William An, Toko Campbell, Cris Brown, Stacy Hurst, Bess Marshall, Tamara Hershey, Virginia Nunes, Miguel López de Heredia, Fumihiko Urano

**Affiliations:** ^1^Division of Endocrinology and Lipid Research, Department of Medicine, Washington University School of Medicine, St. Louis, MO, United States; ^2^Department of Pathology and Immunology, Washington University School of Medicine, St. Louis, MO, United States; ^3^ Medical Scientist Training Program, Washington University School of Medicine, St. Louis, MO, United States; ^4^ Saint Louis University School of Medicine, St. Louis, MO, United States; ^5^ School of Medicine, University of Missouri Kansas City, Kansas City, MO, United States; ^6^Department of Pediatrics, Washington University School of Medicine, St. Louis, MO, United States; ^7^Departments of Psychiatry and Radiology, Washington University School of Medicine, St. Louis, MO, United States; ^8^Molecular Genetics Laboratory, Genes Disease and Therapy Program IDIBELL, L’Hospitalet de Llobregat, Barcelona, Spain; ^9^Genetics Unit, Physiological Sciences Department, Health Sciences and Medicine Faculty University of Barcelona, L’Hospitalet de Llobregat, Barcelona, Spain; ^10^ Centre for Biomedical Network Research on Rare Diseases (CIBERER), Instituto de Salud Carlos III, Madrid, Spain

**Keywords:** wolfram syndrome, WFS1-related disorders, diabetes mellitus, optic atrophy, hearing loss, diabetes insipidus, genotype phenotype correlation

## Abstract

**Objective:** Wolfram syndrome (WFS) is an autosomal recessive disorder associated with juvenile-onset diabetes mellitus, optic atrophy, diabetes insipidus, and sensorineural hearing loss. We sought to elucidate the relationship between genotypic and phenotypic presentations of Wolfram syndrome which would assist clinicians in classifying the severity and prognosis of Wolfram syndrome more accurately.

**Approach:** Patient data from the Washington University International Registry and Clinical Study for Wolfram Syndrome and patient case reports were analyzed to select for patients with two recessive mutations in the WFS1 gene. Mutations were classified as being either nonsense/frameshift variants or missense/in-frame insertion/deletion variants. Missense/in-frame variants were further classified as transmembrane or non-transmembrane based on whether they affected amino acid residues predicted to be in transmembrane domains of WFS1. Statistical analysis was performed using Wilcoxon rank-sum tests with multiple test adjustment applied via the Bonferonni correction.

**Results:** A greater number of genotype variants correlated with earlier onset and a more severe presentation of Wolfram syndrome. Secondly, non-sense and frameshift variants had more severe phenotypic presentations than missense variants, as evidenced by diabetes mellitus and optic atrophy emerging significantly earlier in patients with two nonsense/frameshift variants compared with zero or one nonsense/frameshift variants. In addition, the number of transmembrane in-frame variants demonstrated a statistically significant dose-effect on age of onset of diabetes mellitus and optic atrophy among patients with either one or two in-frame variants.

**Summary/Conclusion:** The results contribute to our current understanding of the genotype-phenotype relationship of Wolfram syndrome, suggesting that alterations in coding sequences result in significant changes in the presentation and severity of Wolfram. The impact of these findings is significant, as the results will aid clinicians in predicting more accurate prognoses and pave the way for personalized treatments for Wolfram syndrome.

## 1 Introduction

Wolfram syndrome (WFS) is an autosomal recessive disorder associated with juvenile-onset diabetes mellitus, optic atrophy, diabetes insipidus, and sensorineural hearing loss (Barrett et al., 1995). Diagnosis of Wolfram syndrome is usually ascertained due to the occurrence of early onset type 1 diabetes mellitus with optic atrophy, which occur in the first decade of life (Barrett et al., 1995; de Heredia et al., 2013). Additionally, central diabetes insipidus and sensorineural deafness occur in the second decade, dilated renal outflow tracts occur in the third decade, and neurological symptoms appear in the fourth decade (Barrett et al., 1995). Patients can also develop a wide range of symptoms including, bladder and bowel dysfunction, temperature regulation defects, gait ataxia, balance deficits, and loss of sense of taste and smell (Barrett et al., 1995; Doty et al., 2017; Samara et al., 2019). WFS symptoms have a detrimental impact on patients’ quality of life and daily functioning (Doty et al., 2017; Samara et al., 2019). The lifespan of patients is expected to be around 30–40 years of age due to respiratory failure caused by brainstem atrophy (Barrett et al., 1995; de Heredia et al., 2013). The prevalence of the disorder is estimated to be 1 in 100,000 in North America and 1 in 770,000 in the United Kingdom (Fraser and Gunn, 1977; Barrett et al., 1995).

Two causative genes, *WFS1* and *CISD2*, have been implicated in the development of Wolfram syndrome. *WFS1* encodes for wolframin, an endoplasmic reticulum (ER) membrane glycoprotein, which plays a role in Ca^2+^ homeostasis and regulates the ER stress response (Takei et al., 2006). Mutations in wolframin lead to ER and mitochondrial dysfunction which cause apoptosis and cell death (Samara et al., 2019). *CISD2* encodes for an ER intermembrane small protein (ERIS) which plays a role in Ca^2+^ homeostasis and mitochondrial function (Samara et al., 2019; Shen et al., 2021). Mutations in *CISD2* were initially described in Jordanian patients with unique phenotypic presentations such as bleeding tendency, defective platelet aggregation with collagen, and peptic ulcer disease (Amr et al., 2007).

Additionally, pathogenic variants in *WFS1* can cause the development of *WFS1*-related disorders involving sensorineural low frequency hearing loss, hearing loss and optic atrophy, cataracts, and an autosomal dominant syndrome characterized by neonatal diabetes, congenital cataracts, sensorineural deafness, hypotonia, intellectual disability, and development delay (De Franco et al., 2017). Dominant *WFS1* variants potentiate ER stress and result in pathophysiology that is distinct and less severe than patients with recessive Wolfram syndrome (De Franco et al., 2017).

The autosomal recessive syndrome has been characterized widely in literature and a variety of pathogenic variants and polymorphisms have been reported to date. In the *WFS1* variants present in the literature, alterations in coding sequences have identified changes including deletions, insertions, nonsense and missense mutations (Khanim et al., 2001). Associations between genotype and phenotype characteristics can suggest the role that gene alterations play in the variability of clinical phenotypes. We sought to elucidate the relationship between genotypic and phenotypic presentations of Wolfram syndrome. Additional information about genotype and phenotype correlations would allow clinicians to classify the severity of Wolfram syndrome more accurately. This could aid in predicting more accurate prognoses and pave the way for personalized treatments for Wolfram syndrome.

The advantages to discovering genotype and phenotype correlations are highlighted in the case of another autosomal recessive disorder, cystic fibrosis. Typing the genotype-phenotype relationship for cystic fibrosis is important as pathogenic variants can alter the expression and function of CFTR via multiple mechanisms (Wang et al., 2014; Veit et al., 2016; Noel et al., 2022). Additionally, the diverse clinical consequences of cystic fibrosis can be attributed to modifier genes and the environment in combination with pathogenic variants (Cutting, 2015; Claustres et al., 2017; Noel et al., 2022). As such, accurate classification of *CFTR* variants, as well as causative *WFS1* variants, is essential in optimizing the treatment of individuals (Noel et al., 2022).

Prior studies have attempted to leverage Wolfram Syndrome genotype information or even protein expression in patient-derived fibroblasts (Smith et al., 2004; Hu et al., 2022; Majander et al., 2022) as predictors or correlates of clinical severity of Wolfram Syndrome. While these studies have successfully correlated biallelic variants (Majander et al., 2022), variants in specific exons (Smith et al., 2004), and low expression of *WFS1* in patient fibroblasts (Hu et al., 2022) with clinical severity of vision impairment and other clinical phenotypes, they all represent smaller cohorts consisting of at most, 37 patients. Consequently, genotype-phenotype correlations made with larger cohorts of patients with Wolfram Syndrome might yield novel insights not just about individual causal variants, but about higher level patterns and “rules” that could provide prognostic information for patients, even for variants that have not yet been observed clinically.

In this study, we aim to classify the range of severity of clinical presentations of autosomal recessive Wolfram syndrome. We classify genetic variants by age of onset, type of genetic variant, and location of variant to identify associations with disease severity. Due to the rare prevalence of Wolfram syndrome, there is fragmented data regarding the correlation between genotype and phenotype presentations. To address this, we compiled patient data from the Washington University International Wolfram Syndrome and *WFS1* Related Disorders Registry and patient data from published case reports compiled in a systematic review by (de Heredia et al., 2013). We performed meta-analysis on these data and found significant correlations between pathogenic variant characteristics and disease severity.

## 2 Materials and methods

### 2.1 Patients

Subjects, and their parents or legal guardians, as appropriate, provided written, informed consent before participating in this study, which was approved by the Human Research Protection Office at Washington University School of Medicine in St. Louis, MO (IRB ID #201107067). Patient data from the Washington University International Registry and Clinical Study for Wolfram Syndrome and patient case reports highlighted in Heredia et al. were analyzed to select for patients with two recessive variants in the *WFS1* gene (de Heredia et al., 2013). Patients were excluded if they lacked genetic information for either of their *WFS1* allele variants. Additionally, records were excluded if they did not have a numerical age of onset for their respective clinical phenotype (diabetes insipidus, optic atrophy, diabetes insipidus, hearing loss). Pathogenic variants were then classified as being either nonsense/frameshift variants or missense/in-frame insertion and deletion variants. For patients with either one or two in-frame variants (missense or in-frame insertion/deletions), their in-frame variant was further classified as transmembrane or not based on whether the amino acid position was in one of the transmembrane domains provided on UniProt (UniProt Consortium, 2023), and age of onset was noted.

### 2.2 Statistical analysis

Statistical analysis was performed by Wilcoxon rank-sum tests with multiple test adjustment applied via the Bonferonni correction. Statistical tests are specified in figure legends. *p* < 0.05 was considered statistically significant. Box plots in all graphs represent the median and quartiles, with whiskers representing 1.5x the interquartile range. Outlier points outside of 1.5x the interquartile range are shown as individual points.

## 3 Results

### 3.1 Onset age of clinical manifestations of Wolfram syndrome

**FIGURE 1:**
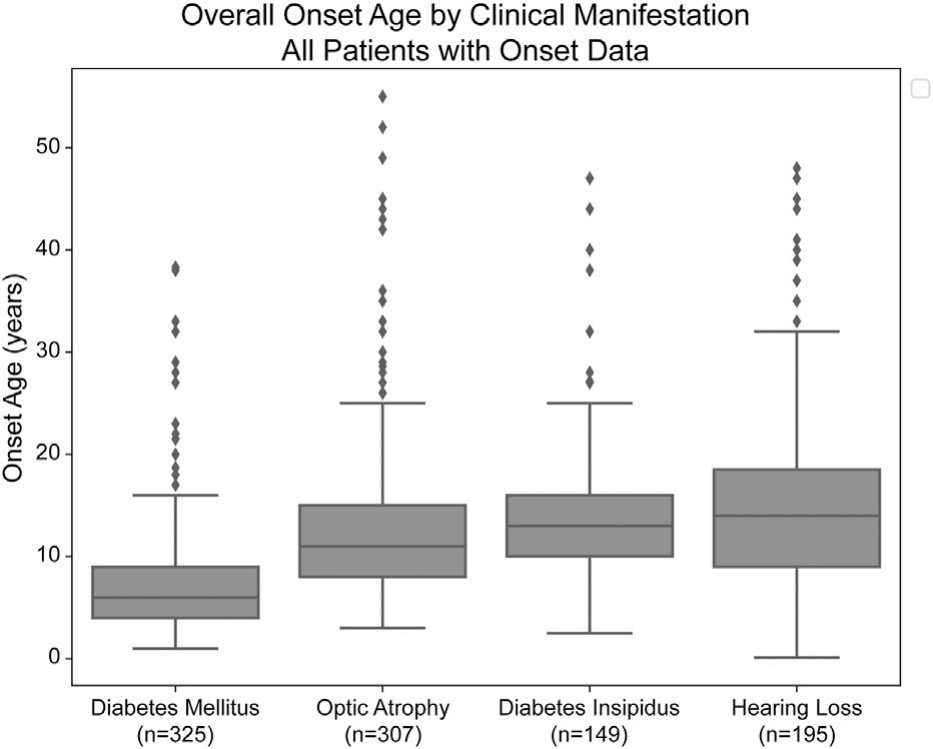
Median age of onset of clinical manifestations of Wolfram Syndrome. Diabetes mellitus, optic atrophy, diabetes insipidus, and hearing loss emerged respectively at median ages of 6.0, 11.0, 13.0, and 14.0 years. Box plots show the following for onset age of each clinical manifestation: inner line, median; box edges, quartiles; whiskers, 1.5x interquartile range; individual points, outliers (defined as outside 1.5x interquartile range). The sample size (n) for each clinical manifestation is shown along the *x*-axis.

Median age of onset of clinical manifestations for the combined patient cohort was calculated for each of diabetes mellitus (DM), optic atrophy (OA), diabetes insipidus (DI), and hearing loss (HL) (Figure 1). The median age of onset was 6.0 years (lower and upper quartiles: 4.0 and 9.0 years, respectively) for diabetes mellitus, followed by 11.0 years (8.0 and 15.0 years) for optic atrophy, 13.0 years (10.0 and 16.0) for diabetes insipidus, and 14.0 years (9.0 and 18.5 years) for hearing loss. The ages of onset are similar to but slightly earlier than those in a similar cohort study of 67 Japanese patients with Wolfram Syndrome, which found median age of onsets to be 8.7 years, 15.8 years, 17.2 years, and 16.4 years for diabetes mellitus, optic atrophy, diabetes insipidus, and hearing loss, respectively (Matsunage et al., 2014).

### 3.2 Nonsense/frameshift *WFS1* alleles exhibit a dose-dependent response on disease severity

**FIGURE 2:**
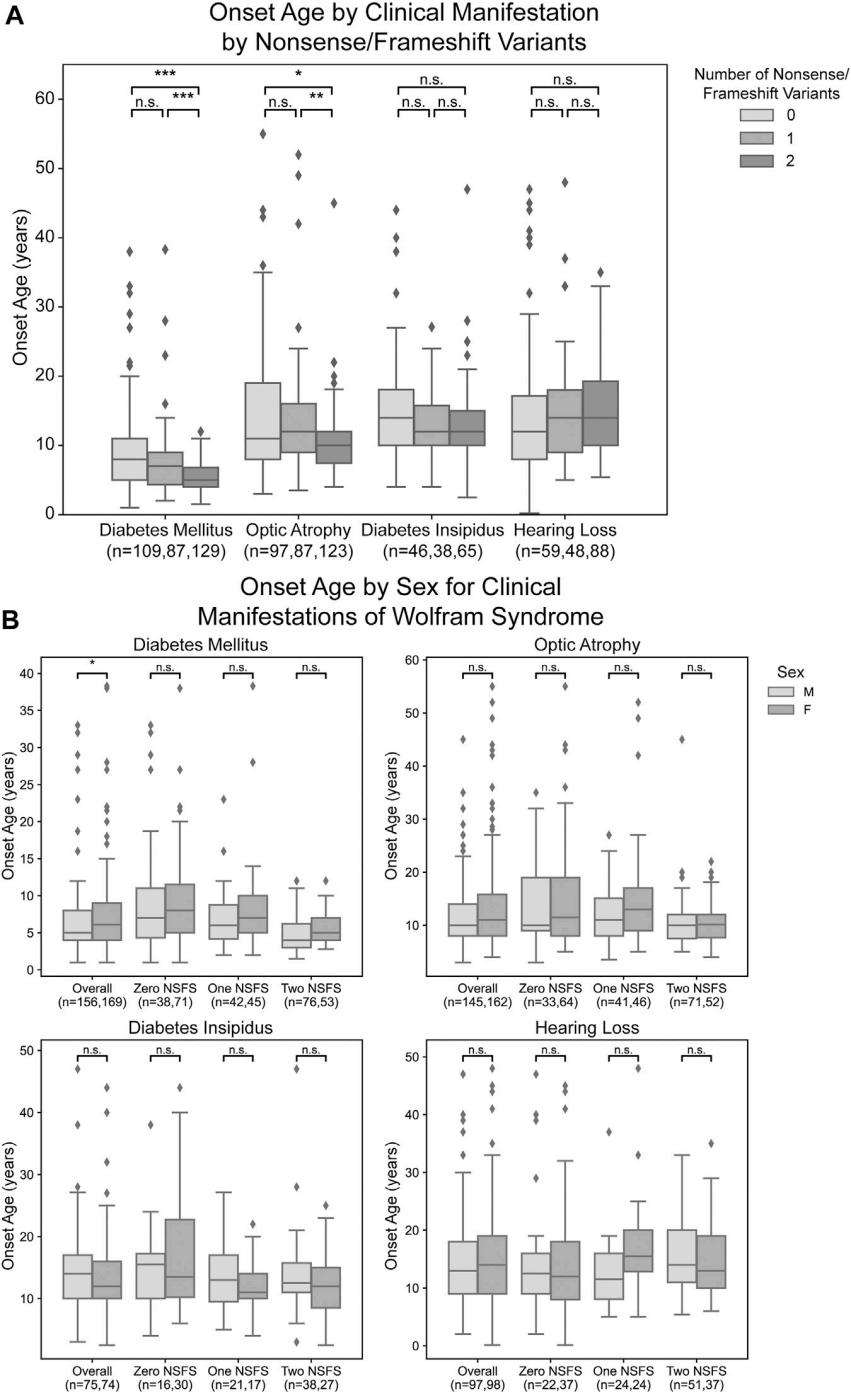
Analysis of genotype-phenotype correlations for number nonsense/frameshift variants. Age of onset of different clinical manifestations by number of nonsense/frameshift (NSFS) variants vs. in-frame missense or insertion/deletion variants for the entire dataset **(A)** and split by patient sex **(B)**. The sample size (n) for each group for each clinical manifestation is shown along the *x*-axis. *, *p* < 0.05; **, *p* < 0.01; ***, *p* < 0.001; n.s., no significance. *p*-values were assigned using Wilcoxon Rank Sum Test with multiple test correction applied via the Bonferroni method. Box plots show the following for onset age of each clinical manifestation within each group: inner line, median; box edges, quartiles; whiskers, 1.5x interquartile range; individual points, outliers (defined as outside 1.5x interquartile range).

For each clinical manifestation of Wolfram syndrome, patients who had information on both alleles as well as numerical age of onset data were further classified based on whether they had zero, one, or two nonsense/frameshift (NSFS) variant alleles. While diabetes insipidus and hearing loss showed no association of onset age with the number of NSFS variants, both diabetes mellitus and optic atrophy demonstrated a dose-effect of number of NSFS variants with respect to age of onset (Figure 2A). Both diabetes mellitus and optic atrophy emerged earliest in patients with two NSFS alleles, followed by patients with one NSFS allele, followed by patients with zero NSFS alleles and only in-frame variants. Diabetes mellitus emerged significantly earlier in patients with two NSFS alleles compared with both zero and one NSFS alleles, and emerged at a median age of 5.0 years (lower and upper quartile: 4.0 and 6.8 years), 7.0 years (4.35 and 9.0 years), and 8.0 years (5.0 and 11.0 years) for two, one, and zero NSFS alleles, respectively. Optic atrophy emerged significantly earlier in patients with two NSFS Alleles compared with both zero and one NSFS alleles, and correspondingly emerged at a median age of 10.0 years (lower and upper quartiles: 7.45 and 12.0 years), 12.0 years (9.0 and 16.0 years), and 11.0 years (8.0 and 19.0 years) for two, one, and zero NSFS alleles. There was not a statistically significant difference in the age of onset between patients with zero NSFS alleles and one NSFS allele for either diabetes mellitus or optic atrophy. The median onset, lower and upper quartiles, number of patients in each category, and statistical test results are shown in Table 1.

**TABLE 1:** Age of onset and Wilcoxon Rank Sums test results for number of nonsense/frameshift variant analysis. (A) Sample size, median, and quartile age of onset information for each clinical manifestation for different numbers of nonsense/frameshift variants. (B) Wilcoxon Rank Sums test *P*- values and Bonferonni adjusted *p*-values for pairwise comparisons of nonsense/frameshift variant numbers. NSFS0: zero nonsense/frameshift variants. NSFS1: one nonsense/frameshift variant. NSFS2: two nonsense/frameshift variants.

Manifestation	Number of NSFS variants	Sample size (n)	Median	Lower quartile	Upper quartile
A					
Diabetes Mellitus	NSFS0	109	8	5	11
Diabetes Mellitus	NSFS1	87	7	4.35	9
Diabetes Mellitus	NSFS2	129	5	4	6.8
Optic Atrophy	NSFS0	97	11	8	19
Optic Atrophy	NSFS1	87	12	9	16
Optic Atrophy	NSFS2	123	10	7.45	12
Diabetes Insipidus	NSFS0	46	14	10	18.0825
Diabetes Insipidus	NSFS1	38	12	10	15.75
Diabetes Insipidus	NSFS2	65	12	10	15
Hearing Loss	NSFS0	59	12	8	17.15
Hearing Loss	NSFS1	48	14	9	18
Hearing Loss	NSFS2	88	14	10	19.25

Manifestation	Comparison	*p*-value	Adjusted *p*-value
B			
Diabetes Mellitus	NSFS2-NSFS0	2.14E-09	2.57E-08
Diabetes Mellitus	NSFS1-NSFS0	5.63E-02	6.76E-01
Diabetes Mellitus	NSFS2-NSFS1	5.07E-05	6.09E-04
Optic Atrophy	NSFS2-NSFS0	4.06E-03	4.87E-02
Optic Atrophy	NSFS1-NSFS0	9.43E-01	1.00E+00
Optic Atrophy	NSFS2-NSFS1	6.42E-04	7.71E-03
Diabetes Insipidus	NSFS2-NSFS0	1.34E-01	1.00E+00
Diabetes Insipidus	NSFS1-NSFS0	1.45E-01	1.00E+00
Diabetes Insipidus	NSFS2-NSFS1	8.30E-01	1.00E+00
Hearing Loss	NSFS2-NSFS0	2.71E-01	1.00E+00
Hearing Loss	NSFS1-NSFS0	5.48E-01	1.00E+00
Hearing Loss	NSFS2-NSFS1	5.40E-01	1.00E+00

One outlier to these findings is emphasized in an exceptional case of typical wolfram syndrome. This patient has a phenotypical clinical diagnosis of typical Wolfram Syndrome with the development of diabetes mellitus at age 5 and optic atrophy at age 8. However, we could only detect a frameshift pathogenic variant in one allele with a corresponding normal allele. Since we could not detect two pathogenic variant alleles, we hypothesized the normal allele was not expressed. This prompted us to look at the expression levels of wild-type and mutated alleles by next-generation sequencing using RNA extracted from induced pluripotent stem cells (iPSC) derived from this patient. Results showed no expression of the wild-type allele, suggesting that there is suppression or methylation in the gene regulatory region of the wild-type allele.

For diabetes mellitus, we observed that male patients had a significantly earlier age of onset when looking at the entire patient cohort with available clinical data (Figure 2B). These differences were not observed at a statistically significant level for any subset of patients based on their number of nonsense/frameshift variants. For other clinical manifestations of Wolfram Syndrome, no statistically significant differences were observed in age of onset between male and female patients, either within the above number of NSFS variant categories or for the cohort overall (Figure 2B).

### 3.3 Missense *WFS1* variants in transmembrane domains are associated with earlier onset of disease

**FIGURE 3:**
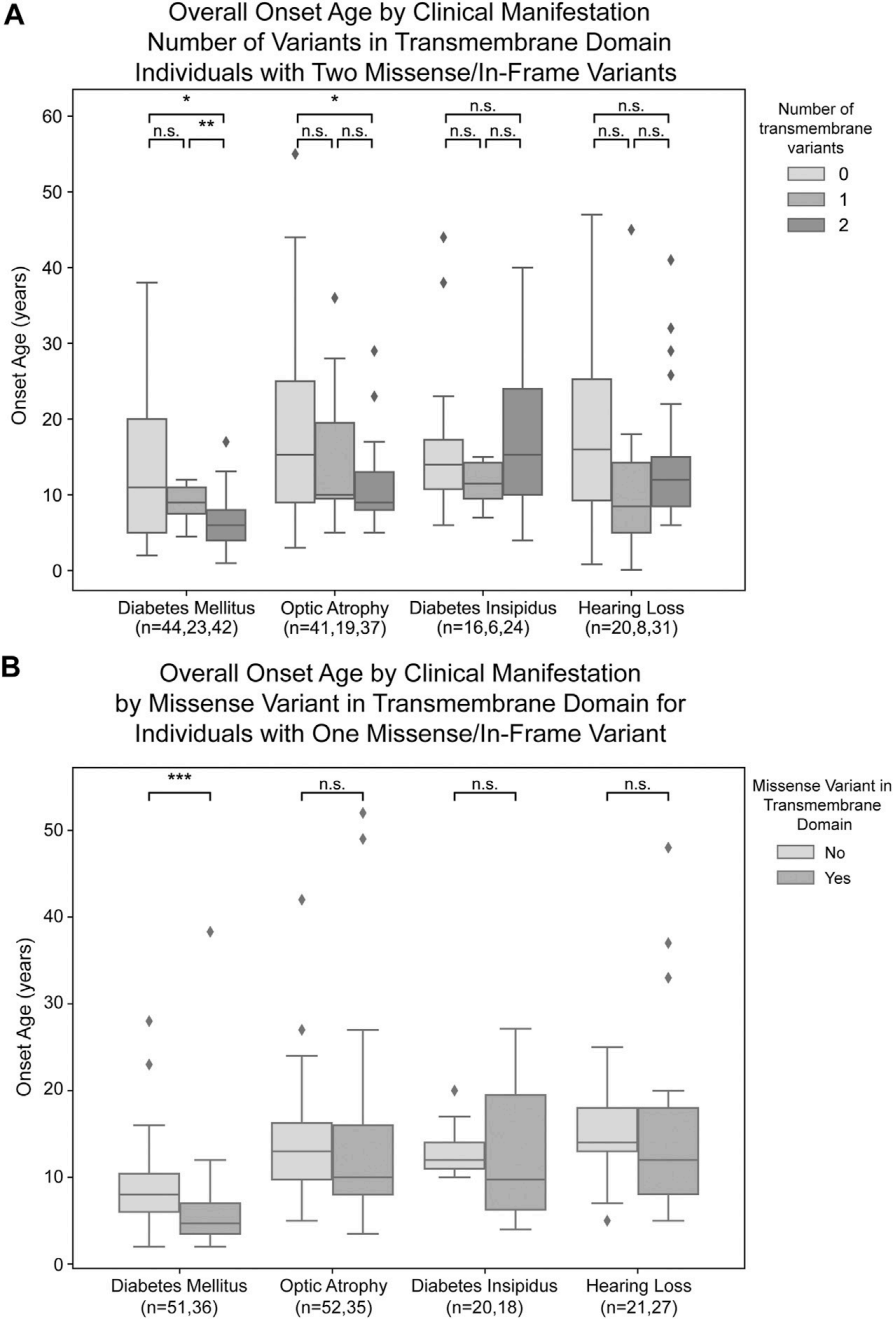
Analysis of genotype-phenotype correlations for in-frame variants by transmembrane/non-transmembrane domain. Age of onset of different clinical manifestations by number of in-frame variants (either missense or in-frame insertion/deletion) in transmembrane domains for patients with two in-frame variants **(A)** and patients with one in-frame variant **(B)**. The sample size (n) for each group for each clinical manifestation is shown along the *x*-axis. *p* < 0.05; **, *p* < 0.01; n.s., no significance. *p*-values were assigned using Wilcoxon Rank Sum Test with multiple test correction applied via the Bonferroni method. Box plots show the following for onset age of each clinical manifestation within each group: inner line, median; box edges, quartiles; whiskers, 1.5x interquartile range; individual points, outliers (defined as outside 1.5x interquartile range).

For missense and in-frame insertion and deletion variants, amino acid position information was matched against the predicted transmembrane domain annotations from UniProt. These variants were then clarified as transmembrane (TM) or non-transmembrane. For each clinical manifestation, the subset of patients previously defined with a) zero NSFS alleles and consequently two in-frame variants and b) one NSFS allele and one in-frame variant were then further classified by whether their in-frame variant(s) were transmembrane or non-transmembrane. Average age of onset for each clinical manifestation was compared by the number of TM alleles (Figure 3A). Patients with two in-frame variants (zero NSFS variants) showed a similar effect of the number of transmembrane variants as seen with the number of NSFS alleles, with the number of in-frame variants which were transmembrane demonstrating a statistically significant dose-effect on age of onset of DM and OA. Diabetes mellitus emerged significantly earlier in patients with two in-frame TM variants compared with both zero and one in-frame TM variants, and correspondingly emerged at a median age of 6.0 years (lower and upper quartiles: 4.0 and 8.0 years), 9.0 years (7.5 and 11.0 years), and 11.0 years (5.0 and 20.0 years) for two, one, and 0 TM variants, respectively. Optic atrophy emerged significantly earlier in patients with two in-frame TM alleles compared with patients that had two in-frame, non-transmembrane variants; however, there was no statistically significant difference in the age of onset between patients with two in-frame TM variants and one in-frame TM variant for optic atrophy. Optic atrophy correspondingly emerged at a median age of 9.0 years (lower and upper quartile: 8.0 and 13.0 years), 10.0 (9.5 and 19.5 years) and 15.3 years (9.0 and 25.0 years) for two, one, and zero in-frame TM variants. The median onset, lower and upper quartiles, number of patients in each category, and statistical test results for patients with two in-frame variants are shown in Table 2(A,B).

**TABLE 2:** Age of onset and Wilcoxon Rank Sums test results for number of transmembrane in-frame variants analysis. A) Sample size, median, and quartile age of onset information for each clinical manifestation for different numbers of transmembrane in-frame variants in patients who have two in-frame variants. (B) Wilcoxon Rank Sums test *p*-values and Bonferonni adjusted *p*-values for pairwise comparisons among numbers of transmembrane in-frame variants in patients who have two in-frame variants. I Sample size, median, and quartile age of onset information for each clinical manifestation for different numbers of transmembrane in-frame variants in patients who have one in-frame variant. (D) Wilcoxon Rank Sums test *p*-values and Bonferonni adjusted *p*-values for pairwise comparisons among numbers of transmembrane in-frame variants in patients who have one in-frame variant. NTM0: zero transmembrane in-frame variants. NTM1: one transmembrane in-frame variant. NTM2: two transmembrane in-frame variants.

Manifestation	Number of in-frame TM variants	Sample size (n)	Median	Lower quartile	Upper quartile
A					
Diabetes Mellitus	NTM0	44	11	5	20
Diabetes Mellitus	NTM1	23	9	7.5	11
Diabetes Mellitus	NTM2	42	6	4	8
Optic Atrophy	NTM0	41	15.3	9	25
Optic Atrophy	NTM1	19	10	9.5	19.5
Optic Atrophy	NTM2	37	9	8	13
Diabetes Insipidus	NTM0	16	14	10.75	17.2775
Diabetes Insipidus	NTM1	6	11.5	9.5	14.25
Diabetes Insipidus	NTM2	24	15.3	10	24
Hearing Loss	NTM0	20	16	9.275	25.25
Hearing Loss	NTM1	8	8.5	5	14.25
Hearing Loss	NTM2	31	12	8.5	15

Manifestation	Comparison	*p*-value	Adjusted *p*-value
B			
Diabetes Mellitus	NTM0-NTM2	1.20E-03	1.44E-02
Diabetes Mellitus	NTM0-NTM1	3.52E-01	1.00E+00
Diabetes Mellitus	NTM1-NTM2	1.26E-04	1.51E-03
Optic Atrophy	NTM0-NTM2	1.54E-03	1.85E-02
Optic Atrophy	NTM0-NTM1	2.33E-01	1.00E+00
Optic Atrophy	NTM1-NTM2	7.90E-02	9.48E-01
Diabetes Insipidus	NTM0-NTM2	7.40E-01	1.00E+00
Diabetes Insipidus	NTM0-NTM1	2.69E-01	1.00E+00
Diabetes Insipidus	NTM1-NTM2	1.78E-01	1.00E+00
Hearing Loss	NTM0-NTM2	2.03E-01	1.00E+00
Hearing Loss	NTM0-NTM1	1.54E-01	1.00E+00
Hearing Loss	NTM1-NTM2	2.44E-01	1.00E+00

Manifestation	Number of in-frame TM variants	Sample size (n)	Median	Lower quartile	Upper quartile
C					
Diabetes Mellitus	NTM0	51	8	6	10.4
Diabetes Mellitus	NTM1	36	4.7	3.5	7
Optic Atrophy	NTM0	52	13	9.75	16.25
Optic Atrophy	NTM1	35	10	8	16
Diabetes Insipidus	NTM0	20	12	11	14
Diabetes Insipidus	NTM1	18	9.75	6.25	19.5
Hearing Loss	NTM0	21	14	13	18
Hearing Loss	NTM1	27	12	8.055	18

Manifestation	Comparison	*p*-value	Adjusted *p*-value
D			
Diabetes Mellitus	NTM0-NTM1	4.33E-05	1.73E-04
Optic Atrophy	NTM0-NTM1	7.32E-02	2.93E-01
Diabetes Insipidus	NTM0-NTM1	4.38E-01	1.00E+00
Hearing Loss	NTM0-NTM1	2.40E-01	9.61E-01

In patients with one in-frame variant and one nonsense/frameshift variant, an in-frame variant in a transmembrane position had statistically significant earlier onset of diabetes mellitus but none of the other clinical manifestations (Figure 3B). For diabetes mellitus, the median age of onset was 4.7 years (lower and upper quartiles: 3.5 and 7.0 years) compared to 8.0 years (6.0 and 10.4 years) for patients with their in-frame variant in a transmembrane position versus those with their in-frame variant in a non-transmembrane position. The median onset, lower and upper quartiles, number of patients in each category, and statistical test results for patients with one in-frame variant are shown in Table 2(C,D).

## 4 Discussion

In the setting of Wolfram syndrome, we sought to explore the associations between genotype and phenotype characteristics to explore the role that gene alterations can play in the variability of clinical phenotypes. It was found that both diabetes mellitus and optic atrophy demonstrated a dose-effect of number of NSFS variants with respect to age of onset. In addition, the number of transmembrane in-frame variants demonstrated a statistically significant dose-effect on age of onset of diabetes mellitus and optic atrophy.

**FIGURE 4:**
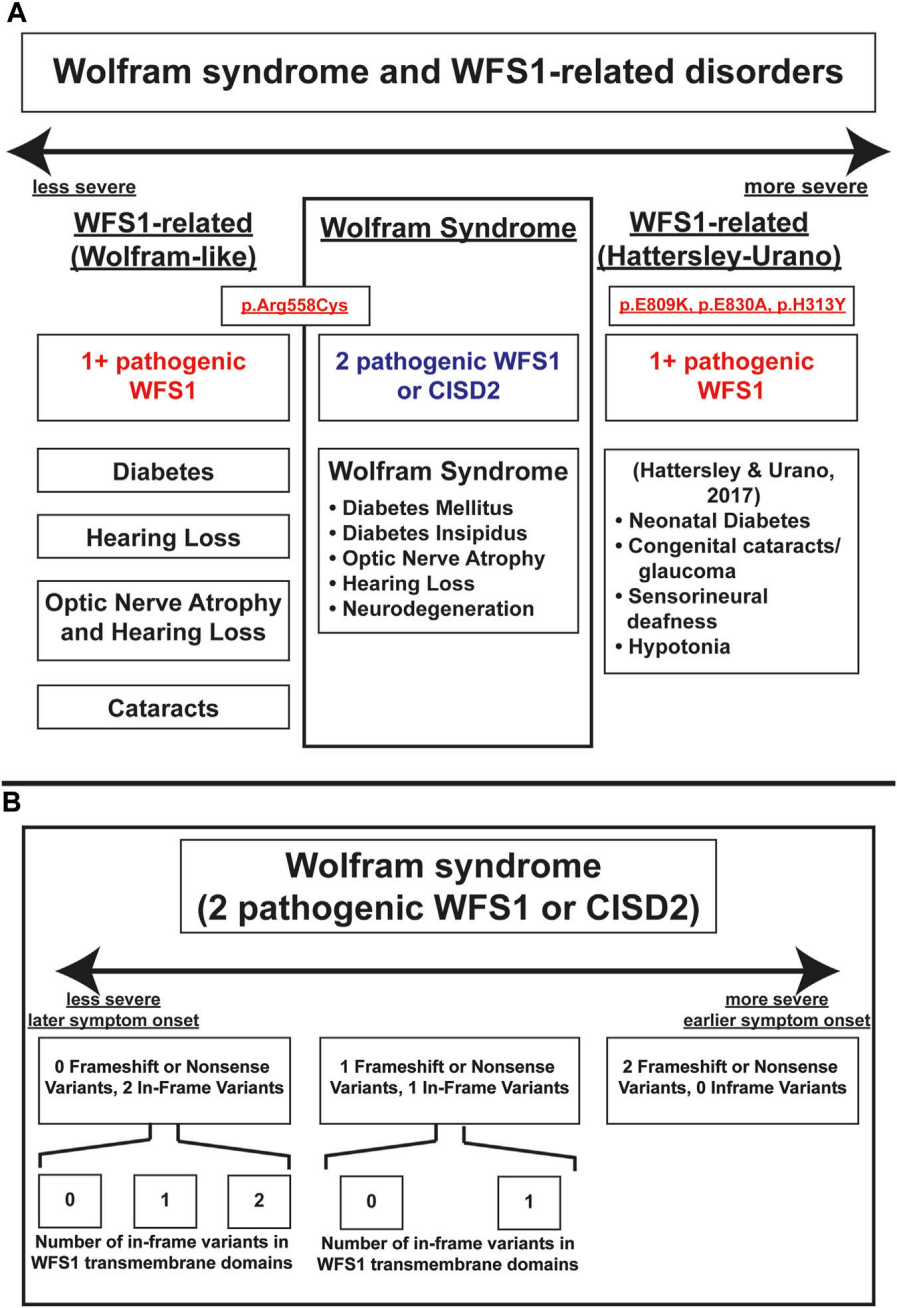
Schema of severity of Wolfram-Syndrome and Wolfram-related disorders. **(A)** Severity and clinical manifestations of Wolfram-related disorders and Wolfram Syndrome. **(B)** Severity of Wolfram Syndrome based on variant number and effect on coding sequence or transmembrane domain positions.

These results highlight principles that are important in understanding the genotype-phenotype relationship in Wolfram syndrome. Firstly, it has been previously suggested that a greater number of variants correlates with earlier onset and a more severe presentation of Wolfram Syndrome (Majander et al., 2022). Autosomal dominant variants have previously been associated with Wolfram-like syndrome, a WFS1-related disorder with generally milder phenotypes and unlike Wolfram Syndrome, no decrease in life expectancy (de Muijnck et al., 2023). An exception to this observed associated between autosomal dominant variants and milder clinical phenotypes has been documented in a subset of patients with heterozygous missense variants in *WFS1* who present with onset of diabetes in the first year of life as well as hypotonia, congenital sensorineural deafness and cataracts (De Franco et al., 2017). In other studies, the relationship between variant characteristics and disease severity has been explored with the discovery of the Arg558Cys variant in Ashkenazi Jewish individuals, which is associated with a milder, late-onset phenotype of Wolfram syndrome including early onset diabetes and reduced penetrance for optic atrophy (Bansal et al., 2018; Wilf-Yarkoni et al., 2021). A schematic illustrating the spectrum of *WFS1*-associated disorders, including their relative phenotypic severity, associated variants, and clinical manifestations is shown in Figure 4A.

Secondly, our results indicate that non-sense and frameshift mutations have more severe phenotypic presentations than missense mutations, as evidenced by optic atrophy emerging significantly earlier in patients with two NSFS variant alleles compared with zero NSFS alleles. Similarly, among in-frame variants, those affecting transmembrane positions are associated with earlier onset of diabetes mellitus and optic atrophy. A schematic of these relationships among variant locations and types is shown in Figure 4B. A similar phenomenon is highlighted in the case of another autosomal recessive disorder, cystic fibrosis. Literature on genotype-phenotype correlations in cystic fibrosis predicts milder phenotypes and better prognosis associated with A455E, a missense variant, compared to ∆F508, a deletion variant (Gan et al., 1995).

The observation that in-frame variants affecting transmembrane domains are associated with earlier onset of disease is intriguing and requires further study and functional characterization. The importance of transmembrane domains in genetic disease has been demonstrated in the context of Pelizeus-Merzbacher disease, an early-onset leukodystrophy defined by mutations of the *PLP1* gene. It has been shown that mutant isoforms of the *PLP1*-encoded proteins PLP and DM20 which disrupt the four endogenous transmembrane domains are fully retained in the endoplasmic reticulum (Dhaunchak et al., 2011). Given the important role of transmembrane domains in membrane anchoring and subcellular trafficking of proteins as well as the known roles of wolframin in the ER stress response and Ca^2+^ homeostasis, characterization of *WFS1* transmembrane variants causing potential localization defects or functional deficits in Ca^2+^ and ER stress tolerance represent intriguing directions for future study.

Similarly, WFS1 protein has been shown to interact with various molecular partners, such as the Sarco endoplasmic reticulum Ca^2+^ ATPase (SERCA) (Zatyka et al., 2015), Sigma 1 Receptor (SIG1R) (Crouzier et al., 2022), Neuronal Calcium Sensor 1 (NCS1) (Angebault et al., 2018), and Activating Transcription Factor 6 (ATF6) (Fonseca et al., 2010), each of which have crucial roles in cellular functions. Therefore, pathogenic variants in the *WFS1* gene could potentially disrupt these interactions and consequently perturb the functions of these molecules. For instance, disruption of WFS1-SERCA interaction could affect calcium homeostasis, while alterations in the WFS1-ATF6 interaction may impair the unfolded protein response, a mechanism vital to handling ER stress. The potential impact of *WFS1* mutations on its interaction with SIG1R and NCS1 could also lead to wide-ranging effects, given their roles in modulating ion channels and neuronal activity, respectively. These possible consequences underscore the need for further research to fully elucidate the complex network of interactions involving *WFS1* and the broader implications of *WFS1* variants.

Lastly, we observed significantly earlier onset of diabetes mellitus in male patients compared to female patients, though we did not observe this trend for optic atrophy or among any of the subsets of patients by their number of nonsense/frameshift variants. This finding of earlier onset diabetes mellitus is consistent with the pathophysiology of Wolfram syndrome involving ER stress and previous evidence that estrogen can mitigate ER stress (Xu et al., 2018). Additionally, in *WFS1* knockout mice, males have been shown to present with more severe phenotypes than females (Abreu et al., 2020). These findings raise the possibility that female patients would present with milder manifestations of Wolfram syndrome, which is supported by our data for diabetes mellitus onset.

The impact of these findings is significant as the results offer contribution to our current understanding of the genotype-phenotype relationship of Wolfram syndrome. These results highlight that alterations in coding sequences result in significant changes in the presentation and severity of Wolfram Syndrome.

The implications of these findings on clinical management of patients with Wolfram Syndrome is another potential direction for future studies. The Wolfram Unified Rating Scale (WURS) has previously been developed and tested for reliability and validity in quantifying disease severity in Wolfram Syndrome patients (Nguyen et al., 2012). Studying the relationship between WURS disease severity scores and variants of different classes such as those explored in this study could be used to make more specific and personalized prognoses for patients with Wolfram Syndrome based on their genotype. Similarly, the development of clinically meaningful biomarkers for Wolfram Syndrome remains an important research goal and would aid clinicians in providing more accurate prognoses of outcomes and disease progression for patients with Wolfram Syndrome. Prior studies have explored *WFS1* expression levels and their correlations with clinical progression (Hu et al., 2022). Similarly, biomarkers for neurodegeneration, such as neurofilament light chain and myelin basic protein, and inflammatory cytokine levels have been shown to be elevated in patients with Wolfram (Abreu et al., 2021; Panfili et al., 2021; Eisenstein et al., 2022). Establishing correlations between these biomarker levels and phenotype severity may eventually pave the way for their validation and use as prognostic tools. This further highlights the need for large patient registries such as those described in this study to provide sufficient statistical power to develop meaningful clinical prognostic tools.

The same variant classes highlighted in this study might represent patient populations that may respond differently to previously-reported potential pharmacological therapies (Lu et al., 2014; Nguyen et al., 2020; Abreu et al., 2021). Further understanding of the mechanisms by which patient *WFS1* variants functionally affect ER and Ca^2+^ stress responses may aid in the development and identification of other potential therapeutic targets and agents, though further studies are needed to explore this area of research.

Although correlations between genotype and phenotype presentations for Wolfram syndrome have been posed in this study, further research is needed to validate these hypotheses.

## Data Availability

The datasets presented in this study, including patient genotype information and clinical data corresponding to age of onset, can be found in the Supplementary Data Sheet S1.
